# The epidemic of Q fever in 2018 to 2019 in Zhuhai city of China determined by metagenomic next-generation sequencing

**DOI:** 10.1371/journal.pntd.0009520

**Published:** 2021-07-15

**Authors:** Mingxing Huang, Jinmin Ma, Jun Jiao, Chunna Li, Luan Chen, Zhongyi Zhu, Feng Ruan, Li Xing, Xinchun Zheng, Mengjiao Fu, Binyin Ma, Chongjie Gan, Yuanchen Mao, Chongnan Zhang, Ping Sun, Xi Liu, Ziliang Lin, Lu Chen, Zhiyu Lu, Dongsheng Zhou, Bohai Wen, Weijun Chen, Xiaolu Xiong, Jinyu Xia

**Affiliations:** 1 Department of Infectious Diseases, the Fifth Affiliated Hospital of Sun Yat-Sen University (SYSU), Zhuhai, China; 2 Guangdong Provincial Key Laboratory of Biomedical Imaging, The Fifth Affiliated Hospital, Sun Yat-sen University, Zhuhai, China; 3 Department of Microbiology, Zhongshan School of Medicine, Sun Yat-sen University, Guangzhou, China; 4 BGI-Shenzhen, Shenzhen, China; 5 State Key Laboratory of Pathogen and Biosecurity, Beijing Institute of Microbiology and Epidemiology, Academy of Military Medical Sciences, 20# Dong-Da-Jie Street, Fengtai, Beijing, China; 6 BGI PathoGenesis Pharmaceutical Technology, BGI-Shenzhen, Shenzhen, China; 7 Zhuhai Center for Disease Control and Prevention, Zhuhai, China; 8 BGI Education Center, University of Chinese Academy of Sciences, Shenzhen, China; Chengde Medical University, CHINA

## Abstract

Q fever is a worldwide zoonosis caused by *Coxiella burnetii* (Cb). From January 2018 to November 2019, plasma samples from 2,382 patients with acute fever of unknown cause at a hospital in Zhuhai city of China were tested using metagenomic next-generation sequencing (mNGS). Of those tested, 138 patients (5.8%) were diagnosed with Q fever based on the presence of Cb genomic DNA detected by mNGS. Among these, 78 cases (56.5%) presented from Nov 2018 to Mar 2019, suggesting an outbreak of Q fever. 55 cases with detailed clinical information that occurred during the outbreak period were used for further analysis. The vast majority of plasma samples from those Cb-mNGS-positive patients were positive in a Cb-specific quantitative polymerase chain reaction (n = 38) and/or indirect immunofluorescence assay (n = 26). Mobile phone tracing data was used to define the area of infection during the outbreak. This suggested the probable infection source was Cb-infected goats and cattle at the only official authorized slaughterhouse in Zhuhai city. Phylogenic analysis based on genomic sequences indicated Cb strains identified in the patients, goat and cattle were formed a single branch, most closely related to the genomic group of Cb dominated by strains isolated from goats. Our study demonstrates Q fever was epidemic in 2018–2019 in Zhuhai city, and this is the first confirmed epidemic of Q fever in a contemporary city in China.

## Author summary

Generally, the clinical diagnosis of acute Q fever, which is caused by *Coxiella burnetii*, is based on serologic methods that detect the presence antibodies produced by the body to fight the infection. However, the lag time between becoming infected and production of antibodies limits early diagnosis using this method. Here, we confirmed an epidemic of human Q fever in Zhuhai, a contemporary city in China, using clinical metagenomic next-generation sequencing (mNGS) and cell phone location data. Our results indicate that Cb*-*infected goats and cattle at the only official authorized slaughterhouse in Zhuhai were the likely infection source for the Q fever epidemic. More importantly, we demonstrate that mNGS is a useful tool for rapid and effective public health responses to acute bacterial infections.

## Introduction

Q fever is a zoonotic infectious disease caused by *Coxiella burnetii* (Cb)[1], a small gram-negative acidophilic bacterium. Acute Q fever typically presents with flu-like symptoms, including high fever, headache, myalgia, and pneumonia, and can develop into severe complications or chronic disease, such as endocarditis and osteomyelitis[2]. Humans are most often infected via Cb-contaminated aerosols while working with infected livestock, mainly goats, sheep and cattle, after an incubation period of 7–32 days[3]. Q fever, described for the first time among abattoir workers in Australia[4], is now recognized as being endemic worldwide[2]. From 2007 through 2010, the Netherlands experienced the largest Q fever epidemic event reported, with over 4,000 identified human cases and 74 deaths[5,6]. Other outbreaks of Q fever have occurred in numerous countries, including Spain, Switzerland, Great Britain, Germany, France[7], the United States[8] and Australia[9]. Although human and animal infections are known to be relatively common in China[10,11], Q fever is not a reportable disease in the country and clinical cases are probably largely unrecognized due to insufficient and inadequate disease surveillance[12].

Early detection of Cb in various samples will promote control of human Q fever and prevent epidemics. Serologic detection of antibodies against Cb is most commonly used in the diagnosis of Q fever[13] because setting a Cb culture is challenging in most clinical laboratories. However, serologic detection is often delayed as blood may not be positive for Cb-specific antibodies until 7–11 days after the primary infection. Thus, molecular detection, such as polymerase chain reaction (PCR), may be a more sensitive approach for early Q fever diagnosis[14]. In recent years, clinical metagenomic next-generation sequencing (mNGS)[15], a novel molecular method that can characterize all DNA or RNA present and identify various microbes in samples, has been applied to identify pathogens, including Cb[16].

Fever of unknown cause is the most common clinical presentation in acute infectious diseases. The pathogens causing fever must be identified as soon as possible to allow effective treatment. We were using the clinical mNGS platform for routine pathogen detection in patients with acute fever of unknown cause at a hospital in Zhuhai[17], when we detected the first Cb-mNGS-positive case in Apr 2018. More cases were detected in the following months, indicating an epidemic of Q fever occurred in Zhuhai. To the best of our knowledge, Q fever epidemic had never been reported before in the contemporary city of China. To validate our postulation, we collected more samples from patients at the hospital for mNGS analysis and laboratory confirmation.

## Materials and methods

### Ethics statement

This study was accordant with the Declaration of Helsinki and approved by the Ethics Committee of the Fifth Affiliated Hospital, Sun Yat -sen University (NO. K226-1), written informed consent was obtained from all study participants.

### Sample collection

From January 2018 to November 2019, patients (n = 2,382) with a symptom of acute fever (above 38.5 ^o^C) for more than 3 days had been treated in the Fifth Affiliated Hospital of SUN Yat-sen University, the biggest division of infectious disease in Zhuhai city. These patients were considered to have acute fever of unknown origin based on the negative results of routine etiological examination, including blood culture, sputum culture, sputum smear for pathogens, influenza screening, Widal’s test, Weil-Felix reaction, cytomegalovirus antibody, and Epstein Barr virus antibody, and ineffective routine antipyretic treatment. Plasma samples from these patients were collected for pathogen detection by mNGS. From December 2018 to March 2019, a total of 218 blood samples from cattle (between 10 and 12 months-old) and goats (between 8 and 12 months-old) were randomly collected by abattoir workers at the slaughterhouse in Zhuhai and used for pathogen detection by mNGS.

### Pathogen identification by metagenomic next-generation sequencing (mNGS)

Plasma samples from the patients underwent mNGS through a PMSEQ product at the BGI PathoGenesis Pharmaceutical Technology Co., Ltd (Shenzhen, China). A sample of 0.16 mL plasma from each patient (or cattle/goat) had DNA extracted using the TIANamp Micro DNA Kit (DP316, TIANGEN Biotech, Jiangsu, China) and following the manufacturer’s recommendations. Extracted cell-free DNA (cfDNA) was quantified by a Qubit 4.0 (Invitrogen, Singapore), and up to 200 ng used for library construction. A DNA library was constructed from cfDNA after end-repair, adapter-ligation, and PCR amplification using the MGI DNA construction kit (MGI Tech Co., Ltd, Shenzhen, China). The constructed library was qualified by Agilent 2100 (Agilent Technologies, Santa Clara, CA) and Qubit 4.0 (Invitrogen) for quality control. The qualified double-stranded DNA library (DNA concentration >1 ng/μL and fragment size ~280 bp) was transformed into a single-stranded circular DNA library through DNA-denaturation and circularization. DNA nanoballs (DNBs) were generated from a single-stranded circular DNA using rolling circle amplification (RCA) and qualified using Qubit 4.0. Qualified DNBs were loaded on the flow cell and sequenced using 50 bp single-end sequencing on the BGISEQ-50 platform (BGI Genomics, Shenzhen, China)[18], which produced at least 20 million reads per sample. A total of eight samples, including one control (HeLa cell) and seven patient samples, were pooled together as one LANE for sequencing. The libraries constructed from cattle or goat plasma samples were also sequenced with the Cb-negative controls.

We first subtracted human sequences from libraries by mapping them to the human reference genome (hg19) using Burrows-Wheeler Alignment[19]. Low-quality sequences (including those with average Phred quality score <20, those with >10% ambiguous bases, and those with low-complexity reads) next were removed using the SOAPnuke software[20]. The remaining data were classified by simultaneous alignment to four microbial genome databases of viruses, bacteria, fungi, and parasites. The databases, downloaded from NCBI (ftp://ftp.ncbi.nlm.nih.gov/genomes/), contain 4,152 whole-genome sequences of viruses, 3,446 bacterial genomes or scaffolds, 206 fungi related to human infection, and 140 parasites associated with human diseases. The number of unique alignment reads was calculated and standardized to obtain the stringently mapped to pathogen species reads number (SDSMRN) and the standardized stringently mapped to pathogen reads number in genus (SDSMRNG). The coverage ratio and depth of each microorganism were calculated using BEDTools[21].

### Serologic analysis

Cb phase I strain Xinqiao and phase II strain Nine Mile II were propagated in embryonated eggs, as previously described[22]. After 6–8 days cultivation, the yolk sacs were harvested and homogenized into cell suspensions. Then, 4 μL of cell suspension was placed on a 12-well multi-test slide (ThermoFisher, Beijing, China) and allowed to air dry. Once dry, the slides were fixed in acetone for 15 min at room temperature. An indirect immunofluorescence assay (IFA) was performed with two-fold serial dilution of plasma from 1:25 to 1:800 in PBS, as previously described[22]. Fluorescein isothiocyanate-conjugated goat anti-human IgG (Proteintech, China), donkey anti-goat IgG (Jackson ImmunoResearch, PA, USA), or goat anti-bovine IgG (Jackson ImmunoResearch) was used to detect the IgGs to Cb. A phase II IgG titer of 1:100 or higher was considered as an index of seropositivity of acute Q fever.

### qPCR Detection

The total genomic DNA was extracted from a plasma sample (200 μL) using the DNeasy Blood & Tissue kit (Qiagen, GmbH, Germany), following manufacturer’s instructions. The DNA sample was eluted from the DNA extraction column with elution buffer (200 μL). DNA were amplified via qPCR with primers *com*1F (5’-AAAACCTCCGCGTTGTCTTCA-3’) and *com1*R (5’-GCTAATGATACTTTGGCAGCGTATTG-3’) and a probe (FAM-AGAACTGCCCATTTTTGGCGGCCA-BHQ-X) targeting the *com*1 gene of Cb. The primers and probe were synthesized by BGI (Beijing, China). The qPCR was performed with a SLAN-96P real-time PCR System (Hongshi, Shanghai, China) using 30 μL reaction volumes consisting of: 2 μL of DNA, 15 μL of 2× PCR Hot-start PCR Mix, 0.6 μL PCR Enzyme Mix, 0.75 μL forward primer (10 μmol/L), 0.75 μL reverse primer (10 μmol/L), 0.375 μL probe (10 μmol/L) and 10.5 μL water. The mixture was kept at 95°C for 10 minutes and then amplified for 40 cycles of 95°C for 15 s and 60°C for 30 s. Fluorescence was recorded during the 60°C phase, and a sample was considered to be Cb-positive if its cycle threshold (CT) value was lower than the mean CT value minus 6 standard deviations of samples from healthy people.

### Traceback of patients by mobile phone location

To identify the probable location of infection for human Q fever cases, the location history of patients’ mobile phones was used. After informed consent, the mobile phone location history of each patient was collected for the 30 days immediately before the patient came to the hospital. The mobile phone data downloaded from mobile operators and analyzed based on the user’s natural attributes and location information, time spent in each location was recorded as the working and living places (working time: 10:00–17:00 is recorded as working places; residence time: 17:00–10:00 include sleep time: 1:00–6:00 are recorded as living place). The cumulative duration time of all patients at each location was summed, and results coded using color: the darker the color, the higher the number of patients who had been to the region.

### Genetic analysis

All Cb mNGS reads from 55 Cb positive samples with detail clinical information were merged and then mapped to whole-genome Cb reference sequences downloaded from GenBank (ftp://ftp.ncbi.nlm.nih.gov/genbank/). The reference with the highest coverage of the genome was used as a trust contig parameter to do the whole-genome assembly by software SPAdes 3.13.0[23] with kmer lengths ranging from 22 to 44. Whole-genome comparisons were performed with nucmer 3.9.4 in the MUMmer package[24]. Finally, a matrix of single-nucleotide polymorphisms (SNPs) was calculated by MUMmer for phylogenetic analyses using the maximum likelihood method in the Molecular Evolutionary Genetics Analysis software (MEGA) version 6[25].

## Results

### Pathogen identification by metagenomic next-generation sequencing

**Fig 1:**
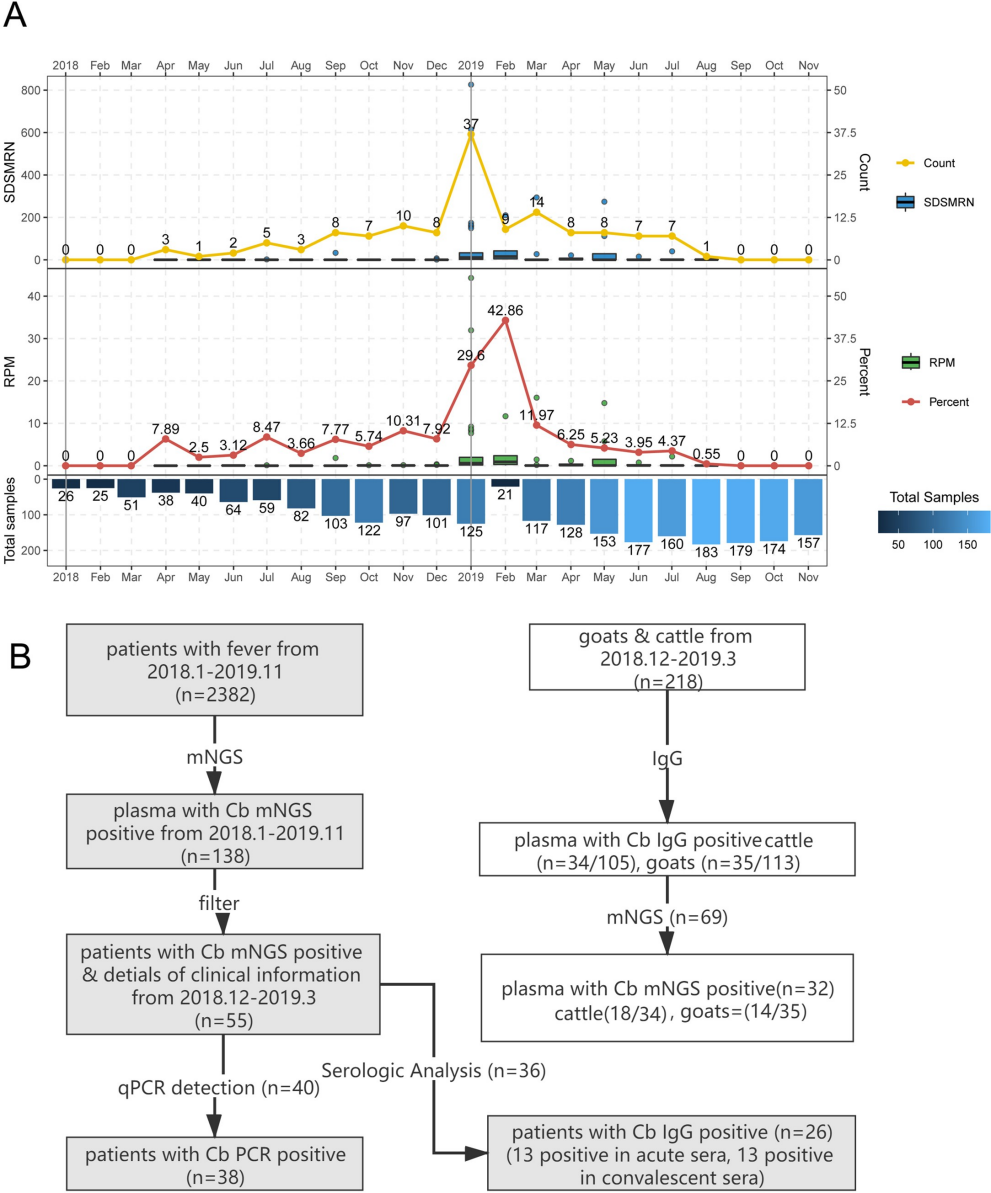
Two years monitoring for Q fever cases and pathogen identification process by mNGS. A. The number and percentage of Q fever cases every month. SDSMRN: Stringently Mapped to pathogen species Reads Number, RPM: Reads per Million, Percent: (number of Q fever patients)/ (number of the patients with unknown fever). The blue bar indicated the sample number for mNGS testing. B. Identification process of Q fever pathogen in patients and animals.

**Fig 2:**
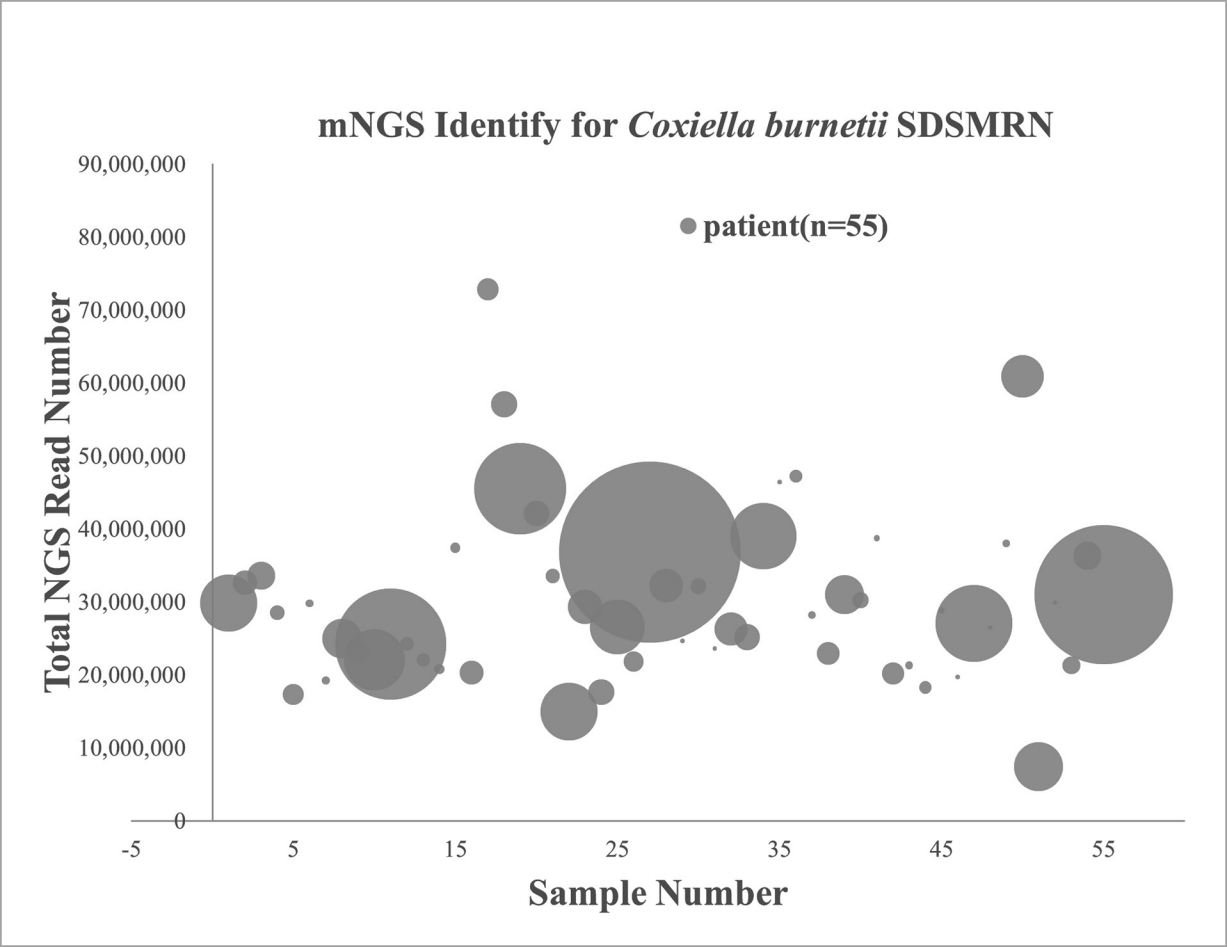
Metagenomic next-generation sequencing for identifying *C*. *burnetii* in patients. The total mNGS read number for each sample. Each circle represents an individual sample and the diameter represents the SDSMRN value.

From January 2018 to November 2019, a total of 2,382 patients with acute fever of unknown origin presented at the hospital in Zhuhai. Samples from 138 patients (5.8%) contained genomic DNA of Cb and no genomic DNA of other pathogens, suggesting these patients had Q fever. Among these Q fever cases, 78 cases (56.5%) presented from November 2018 to March 2019, and 60 cases scattered among the remaining months in 2018–2019 (Fig 1A), indicating a main outbreak of Q fever from November 2018 to March 2019. Therefore, the 55 Q fever cases with detailed clinical information that occurred during the outbreak period were chosen for further study (Fig 1B). Pathogen identification was completed within 26 hours after the plasma samples arrived in the laboratory. An average of 29 million mNGS reads were generated by the BGISEQ-50 sequencer for each patient sample, and 1–1,632 (average 106.76, n = 55) SDSMRN sequences were detected (Fig 2).

### Patient features

The detailed clinical characteristics of Cb-mNGS-positive patients and Cb-mNGS-negative patients during the outbreak period (from November 2018 to March 2019) are shown in Tables 1 and S1. The levels of infectious markers, procalcitonin, C-reactive protein (CRP), and erythrocyte sedimentation rate (ESR) in both Cb-mNGS-positive and Cb-mNGS-negative patients were higher than the upper limit of normal. While the percent of male patients and patients with fatty liver disease in Cb-mNGS-positive group were significantly higher than those in Cb-mNGS-negative group.

**Table 1:** Clinical characteristics of the patients with acute Q fever positive Cb-mNGS-positive and Cb-mNGS-negative. * Significantly different between Cb-mNGS-positive and Cb-mNGS-negative (t-test, *P*<0.05). ** Significantly different between Cb positive and negative (2×2 CHITEST *P*<0.05).

Variable	Cb mNGS positive Patients (n = 55)	Cb mNGS negative Patients (n = 264)
Male sex (%)**	51/55 (92.7%)	179/264 (67.8%)
Age (years)*	43.18±11.17	47.82±16.64
White cell count (10^9^/L)*	5.966±1.816	6.89±3.12
Blood platelet count (10^9^/L)	164.5±59.01	183.69±93.42
Procalcitonin (ng/mL)	1.484±1.642	1.63±2.11
C-reactive protein (mg/L)	78.08±44.62	76.50±51.28
Erythrocyte sedimentation rate (mm/h)*	23.06±15.99	31.58±24.95
ALT (U/L)*	122.6±83.06	84.42±63.48
AST (U/L)*	94.82±65.19	64.48±55.70
CK (U/L)	216.9±232.6	203.76±249.04
CK-MB (U/L)	9.320±3.484	11.96±11.36
Creatinine (μmmol/L)*	95.42±82.31	73.96±57.65
Fatty liver (yes/no)(%)**	17/55(30.9%)	41/264 (15.53%)
Hepatitis (yes/no)(%)	3/55(5.45%)	8/264 (3.03%)
Hepatic cyst (yes/no)(%)	3/55(5.45%)	6/264 (2.27%)
Hepatic hemangioma (yes/no)(%)	3/55(5.45%)	16/264 (6.06%)

### qPCR detection and serologic analysis of patient samples

Using Cb-specific qPCR, 38 of 40 plasma samples tested from the 55 Cb-mNGS-positive patients were positive, with average CT values <31 (S1 Table). As controls, the average CT values of plasma samples from convalescent patients and Cb-mNGS-negative patients were≥33.

Plasma samples from 36 of the 55 patients were tested for Cb phases I and II IgGs in an IFA analysis (Table 2 and S1 Fig). The overall seropositivity rate of these patients was 72.2% (26/36). Among these 26 patients, 13 patients (50.0%, 13/26) had paired-plasma samples (acute and convalescent plasma) changing more than four times in Cb phase II IgG titers, and the other 13 patients (50.0%, 13/26) were diagnosed as having acute Q fever based on the results of the primary blood samples (a phases II IgG titer of 1:100 or higher).

**Table 2:** The Cb-specific IgG titers of the patients detected by IFA.

Patient	In-patient date	Out-patient date	Age	Sex	Acute plasma		Convalescent plasma		Serum-positive
			(years)		Phase I IgG	Phase II IgG	Phase I IgG	Phase II IgG	
1	2018/12/31	2019/1/10	42	male	<25	<25	100	100	P
2	2019/1/1	2019/1/10	35	male	<25	100	≥800	≥800	P
3	2018/12/31	2019/1/15	45	male	<25	<25	400	400	P
4	2018/12/31	2019/1/5	57	male	100	100	≥800	≥800	P
5	2018/12/31	2019/1/5	47	male	<25	<25	100	<25	P
6	2019/1/6	2019/1/12	53	male	<25	<25	400	400	P
7	2019/1/3	2019/1/10	43	male	<25	<25	200	200	P
8	2019/1/7	2019/1/14	52	male	<25	<25	400	≥800	P
9	2019/1/7	2019/1/14	40	male	<25	<25	200	200	P
10	2019/1/7	2019/1/10	51	male	<25	<25	400	400	P
11	2019/1/10	2019/1/15	59	male	<25	<25	100	400	P
12	2019/1/13	2019/1/18	43	male	<25	100	400	≥800	P
13	2019/1/11	2019/1/13	54	male	<25	<25	≥800	≥800	P
14	2019/1/13	2019/1/17	43	male	400	400	NA	NA	P
15	2019/1/14	2019/1/18	30	male	200	200	NA	NA	P
16	2019/1/10	2019/1/18	52	male	100	100	NA	NA	P
17	2019/1/15	2019/1/28	42	male	100	200	NA	NA	P
18	2019/1/15	2019/1/21	29	male	<25	100	NA	NA	P
19	2019/1/14	2019/1/16	37	male	100	100	NA	NA	P
20	2019/1/14	2019/1/18	33	male	<25	100	NA	NA	P
21	2019/1/14	2019/1/19	41	male	<25	200	NA	NA	P
22	2019/1/16	2019/1/18	40	male	400	400	NA	NA	P
23	2019/1/20	2019/1/30	46	male	200	200	NA	NA	P
24	2019/1/19	2019/1/24	57	male	100	100	NA	NA	P
25	2019/1/18	2019/1/22	53	male	100	<25	NA	NA	N
26	2019/1/18	2019/1/22	22	male	200	200	NA	NA	P
27	2019/1/19	2019/1/24	55	male	400	400	NA	NA	P
28	2019/1/26	2019/1/29	39	male	<25	<25	NA	NA	N
29	2019/1/29	2019/2/2	46	male	<25	<25	NA	NA	N
30	2019/1/30	2019/2/2	27	male	<25	<25	NA	NA	N
31	2019/1/30	2019/2/1	26	male	<25	<25	NA	NA	N
32	2019/1/29	2019/2/1	31	male	<25	<25	NA	NA	N
33	2019/1/30	2019/2/3	35	male	<25	<25	NA	NA	N
34	2019/1/30	2019/2/3	49	male	<25	<25	NA	NA	N
35	2019/1/30	2019/2/2	36	male	<25	<25	NA	NA	N
36	2019/1/30	2019/2/2	33	male	<25	<25	NA	NA	N

P, positive; N, negative; NA, not collected.

### Epidemic transmission of *C*. *burnetii*

**Fig 3:**
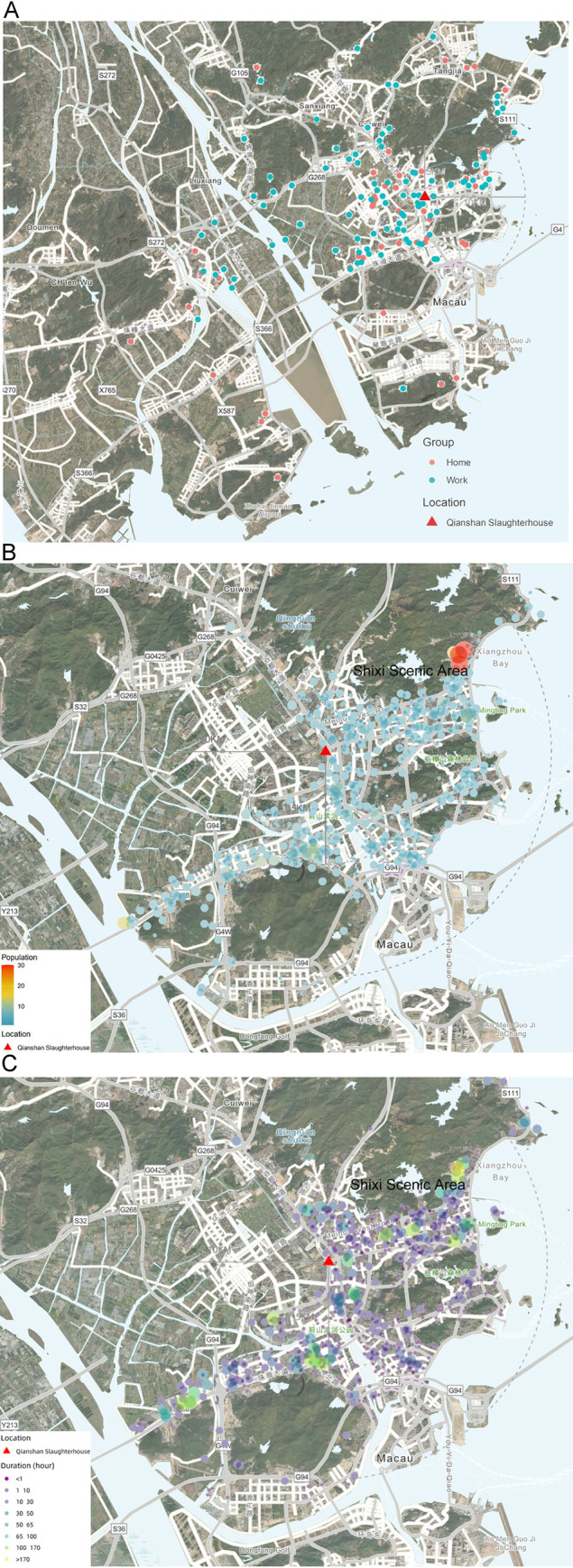
Cell phone positioning for patient tracking. A, the distribution of places where the patients stayed for the longest time. Red color represents home time and blue color represents work time. B. The cumulative numbers of patients found in each location (sleep time removed). Darker (more blue) colors indicates area was more likely workplace location of patients. Red color highlights areas with higher numbers of patients. C. Hours spent in each location by patients (sleep time removed). Darker colors indicate greater cumulative duration of patients at that location. (Source: https://ngmdb.usgs.gov/mapview/?center=113.436,22.221&zoom=12).

Mobile phone location data indicated that all patients were located in Zhuhai city from December 2018 to March 2019 and that most were living near a slaughterhouse (the only officially-authorized slaughterhouse in Zhuhai) and a Fresh Meat Wholesale Center (red triangle in Fig 3A) in the Xiangzhou District (Fig 3A). The cumulative time data showed that most patients lived within 5 km of the slaughterhouse (Fig 3B). Some population hotspots were observed in public resorts, such as the Shixi Scenic Area and Sports center. The cumulative time of patients at each location (Fig 3C) is similar to that of the cumulative numbers of patients shown in Fig 3B. Using duration, more hotspots of >30 hours were observed near the slaughterhouse (Fig 3C). The biggest hotspot still appeared around the Shixi Scenic Area. These analyses suggested that the abattoir might be the source of infection, and that these public locations were associated with a high risk of Cb transmission in the area. Furthermore, in a questionnaire conducted by the hospital, all patients traced by cell phone confirmed they had been to the entertainment restaurant near the slaughterhouse. Thus, samples from goats and cattle at the slaughterhouse were collected for Cb detection and IgG testing.

### *C*. *burnetii* in livestock

Blood samples were collected from cattle and goats at the slaughterhouse within the region patients frequented. A total of 218 blood samples from dairy cattle and goats were tested by IFA with both Cb phases I and II antigens. The seroprevalence rates in cattle and goats were 32.4% (34/105) and 30.9% (35/113), respectively. Detailed information is provided in S2 Table.

**Fig 4:**
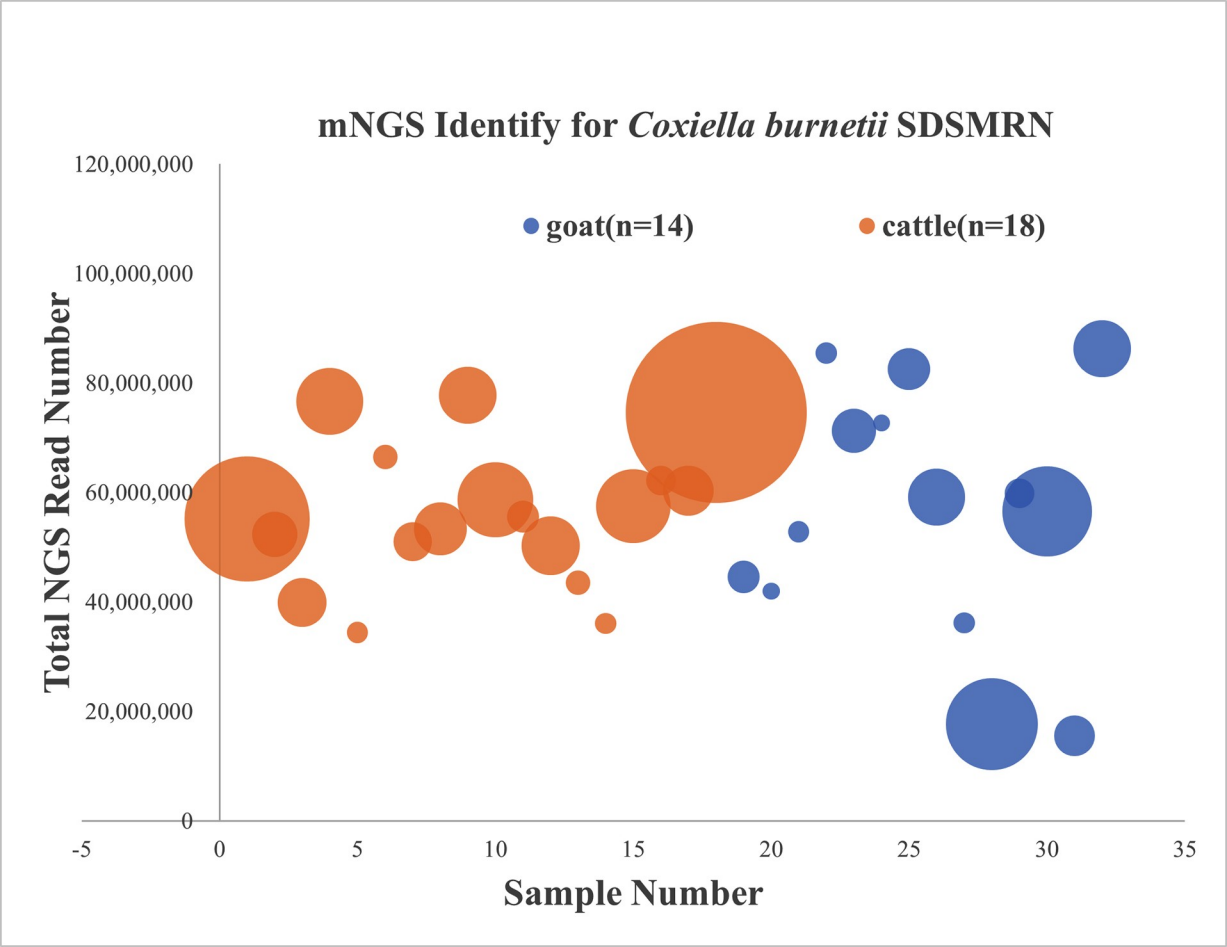
Metagenomic next-generation sequencing for identifying *C*. *burnetii* in livestock. The total mNGS read number for each sample. Each circle represents an individual sample and the diameter represents SDSMRN value. Color indicates animal type: yellow for cattle, blue for goats.

The seropositive samples from goats and cattle were assayed by mNGS, and SDSMRN sequences were detected in 46.4% of the seropositive animals (14 goats and 18 cattle from a total of 69 animals). An average of 55 million mNGS reads were generated for each animal, with 2–57 (average = 15.5, n = 14) and 3–220 (average = 32.11, n = 18) SDSMRN sequences detected in goats and cattle, respectively (Fig 4). The SDSMRN of the cattle (*P* = 0.0027, t-test) and goats (*P* = 0.0024, t-test) were significantly lower than that of the patients, but there was no difference between cattle and goat SDSMRN (*P* = 0.41, t-test).

### Genomic characteristics of strains

**Fig 5:**
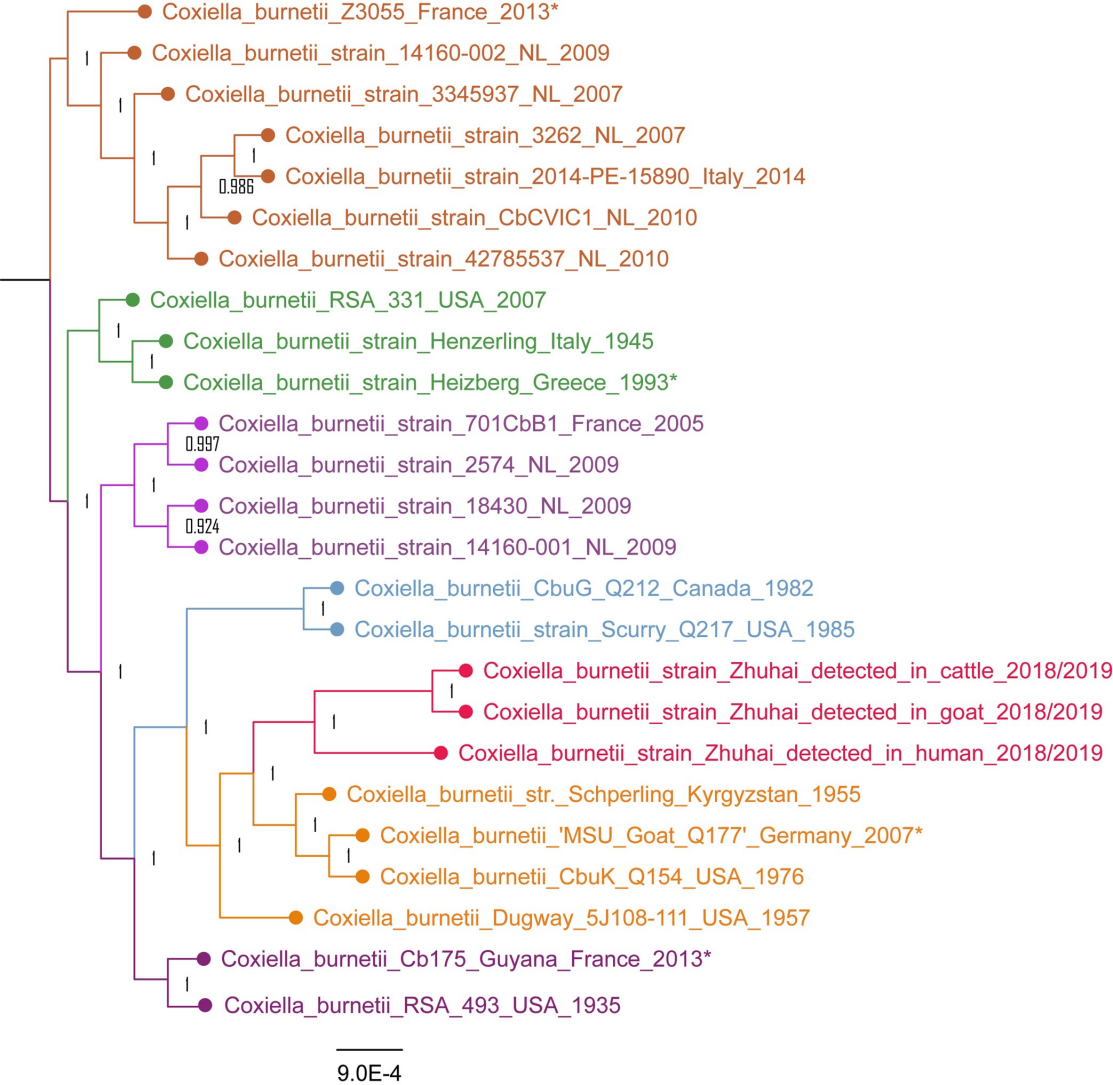
SNP based phylogenetic analysis of the *C*. *burnetii* Zhuhai strains. Zhuhai strains from patients, goats, and cattle form a single branch highlighted in red color. *indicates year when strain was isolated or detected.

All Cb mNGS reads of the patients, cattle, and goats were assembled to obtain partial genomes. The three partial genomes were compared to the 22 complete genomes for Cb from GenBank, based on a total of 33,466 SNP sites. A maximum likelihood phylogenetic tree for Cb strains was created based on the SNP signatures. According to the phylogenetic tree, the strains detected in cattle or goats in Zhuhai are close to each other, and grouped with the strain detected in the patients in Zhuhai, to form a single branch that is associated with a branch that includes the strains Schoperling_Kyrgyzstan_1955, MUS_Goat_Q177_Germany_2007, and CubK_Q154_USA_1976 (Fig 5).

## Discussion

Rapid and effective public health responses to acute bacterial infection require ongoing and timely detection and characterization of the causative pathogens. Conventional pathogen detection methods may take several days to weeks and frequently fail to detect pathogens present. mNGS is a high-throughput sequencing method that can directly detect nucleic acid sequences from DNA of unknown origin in clinical samples. The nucleic acid sequences can then be analyzed using bioinformatics to identify pathogens present. This overcomes many of the limitations of culture methods, targeted nucleic amplification tests, and serologic assays of pathogens[26]. As a novel diagnostic tool, mNGS has been used for the identification of various pathogens in clinical samples (tissues, CSF, or plasma) in an unbiased, simultaneous, and direct manner, which is useful for the rapid detection of difficult-to-culture microbes, such as Cb[27,28], or direct detection of a novel pathogen such as the SARS-CoV-2[29] that emerged in human populations at the beginning of 2020 around the world. The application of continuous mNGS monitoring platforms will help identify pathogens earlier in the initial stages of epidemic outbreaks.

In China, human Q fever was initially reported in 1950, and subsequently, sporadic human Q fever cases and several small outbreaks of Q fever have occurred in leather factories and goat/sheep farms throughout the country[30]. In the present study, the epidemic of acute Q fever in Zhuhai city, China was detected with mNGS monitoring. In patients presenting with a fever of unknown origin, 5.8% were diagnosed as having Q fever based on Cb genomic DNA detected by mNGS. The Cb-positive results were verified by Cb-specific IFA and/or qPCR, demonstrating that mNGS is a reliable and effective method of diagnosing Q fever in the laboratory. Among these Q fever cases, 78 cases (56.5%) presented from November 2018 to March 2019, suggesting an outbreak of Q fever during time in Zhuhai city. In the current study, we focused on the samples collected from these 5 months.

Geographic Information System (GIS) and spatial epidemiological methods can provide a basis for identifying hotspots and epicenters of infection during the investigation of epidemic diseases. Based on the analysis of mobile phone location 30 days before hospitalization, all patients from December 2018 to March 2019 were located in Zhuhai city. In particular, near the slaughterhouse was targeted as a high-risk infection area, where patients dwelt and appeared at high frequency. The Shixi Scenic Area, identified as the largest hotspot, was implicated as an epidemic outbreak area. The successful application of this GIS method reveals clear benefits of this technique for use in COVID-19 pandemic control in China.

Q fever is a zoonosis caused by Cb, with domestic ruminants (goats, sheep, and cattle) the main source of outbreaks in humans through inhalation of Cb-contaminated aerosols[31]. Livestock is often sub-clinically infected, but naïve small ruminants may present reproductive disorders, including abortion, premature delivery, stillbirth, and weak offspring[32]. Livestock shed Cb in high numbers in birth products[33], which have been linked to subsequent human Q fever outbreaks because birth products are heavily contaminated and can easily contaminate the environment. A few Cb specimens are sufficient to cause infection and produce the clinical disease in humans[34,35]. A systematic literature research of putative drivers of Cb geographic dispersal, indicate that the highest infection risk occurs within 5 km of the source. Wind speed or direction, spreading of livestock products, and stocking density may contribute to the Cb environmental infection risk[36].

Seroprevalence was 31.0% in goats and 32.4% in cattle from the slaughterhouse located in the infection area. SDSMRN sequences from Cb were detected in 46.4% of the seropositive animals. These results demonstrated that some of the goats and cattle at the slaughterhouse had experienced Cb infection and suggested that these infected livestock might be the source of this epidemic of human Q fever. When Cb infection appears in goats or cattle at the slaughterhouse, the control measures for biosafety must be quickly implemented to protect workers who are at risk and prevent propagation of the infection. Therefore, samples from the environment and from the workers at the slaughterhouse need to be collected to confirm this transmission route of Q fever.

According to a phylogenetic tree based on the SNP signatures, the strains detected in patients, cattle, and goats in Zhuhai are close to one another, forming a single branch that is mostly closely connected to Schoperling_Kyrgyzstan_1955, MUS_Goat_Q177_Germany_2007, and CubK_Q154_USA_1976. Six genomic groups (GGs) of Cb have been proposed by restriction endonuclease digestion patterns[37] and confirmed by multiple-locus variable number tandem repeat analysis (MLVA)[38] and multispacer sequence typing (MST)[39]. In the phylogenetic analysis of 76 genomes of Cb based on SNP signatures, the Cb strains Schoperling_Kyrgyzstan_1955, MUS_Goat_Q177_Germany_2007, and CubK_Q154_USA_1976 were grouped in GG IV[40]. Therefore, Zhuhai strains identified in this study also belong to the genomic group GG IV, which is dominated by multi spacer typing (MST) 8. This genotype has been linked to goats[41], suggesting that the Cb strains identified in the patients in this epidemic of human Q fever came initially from Cb-infected goats in Zhuhai.

## Conclusion

Patients with acute fever of unknown origin in the city of Zhuhai, China were identified as having Cb infection through mNGS pathogen detection. The majority of plasma samples from Cb-infected patients were positive in the Cb-specific qPCR and/or IFA assay. Meanwhile, Cb was also detected in the samples of both goats and cattle at a slaughterhouse located in the infection area identified by GIS through patients’ cell phone location data. The genomic sequence analysis of Cb showed that the strains detected in the patient, goat, and cattle samples in Zhuhai form a single breach that is most related to Cb genomic group IV, which contains strains isolated from goats worldwide. Our study demonstrates that human Q fever was epidemic in Zhuhai city and that the infection source was probably the Cb-infected goats and cattle from the only official authorized slaughterhouse in Zhuhai city. This epidemic of human Q fever in a contemporary city is the first confirmed in China.

## Supporting information

S1 Fig Representative pictures of the IFA test for Cb-mNGS-positive patients. (TIF)

S1 Table The detailed clinical information of Cb-mNGS-positive patients and Cb-mNGS-negative patients at a hospital in Zhuhai. (XLSX)

S2 Table The seroprevalences in cattle and goats collected from a slaughterhouse in Zhuhai. (XLSX)
