# Supplementary figures and images for: The epidemic of Q fever in 2018 to 2019 in Zhuhai city of China determined by metagenomic next-generation sequencing
Source: PLoS Negl Trop Dis. 2021 Jul 15;15(7):e0009520. doi: 10.1371/journal.pntd.0009520 (PMC8282036; doi:10.1371/journal.pntd.0009520)

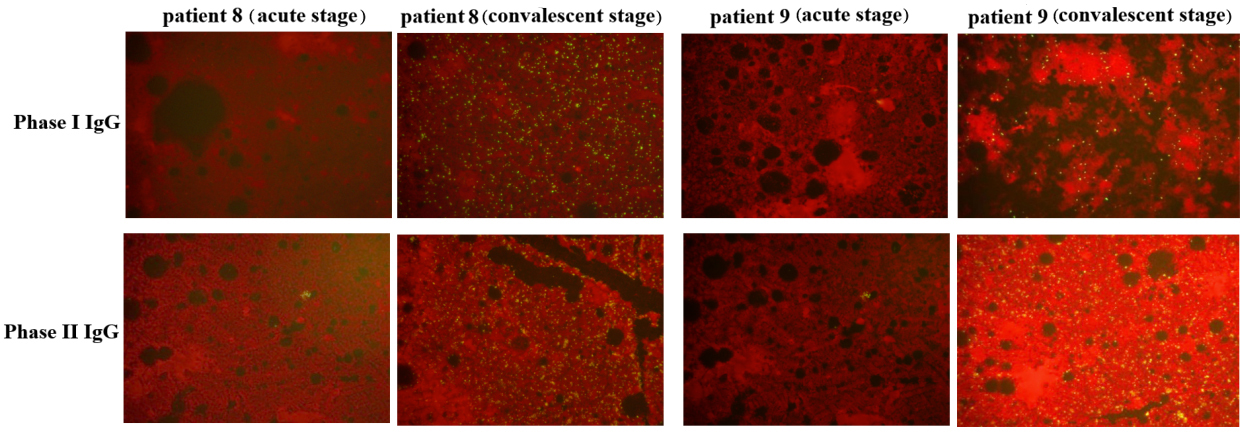

Supplement: S1 Fig — (TIF) [file pntd.0009520.s001.tif]
